# Editorial Comment to First report of testis‐sparing surgery for sertoliform cystadenoma: case presentation and review of literature 

**DOI:** 10.1002/iju5.12378

**Published:** 2021-09-20

**Authors:** Teppei Takeshima

**Affiliations:** ^1^ Department of Urology, Reproduction Center Yokohama City University Medical Center Kanagawa Japan

Orchiectomy is the gold standard for managing testicular tumors. According to the American Urological Association guideline, patients with a suspected malignant testicular lesion and a normal contralateral testis should undergo radical inguinal orchiectomy, instead of testis‐sparing surgery (TSS).^1^ TSS may be offered as an alternative for treating testicular tumors in highly selected patients having tumors of diameter 2 cm or less who wish to preserve testicular function or whose testicular function is likely to be substantially impaired by orchiectomy.^1^ However, TSS is controversial for cases that are strongly suspected to be benign.

Sertoliform cystadenoma is a very rare benign tumor originating from the rete testis. Ultrasound findings show a cystic mass in many cases, but sometimes a solid mass is observed with an epididymal cyst.^2^ If malignancy cannot be completely ruled out after examination with various modalities, orchiectomy is still considered preferable. In particular, given the rarity of sertoliform cystadenoma, it may be difficult to definitely rule out malignancy preoperatively. However, when the mass is obviously benign and no malignant findings on rapid intraoperative pathology after ischemia of the testicular vessels exist, TSS with tumor enucleation with a wide enough margin may be considered. Preoperative evaluation of testicular function, including analysis of semen, serum testosterone levels, and gonadotropin levels, is advised when considering the possibility of conversion to orchiectomy.

In this case, the tumor was judged to be benign because there was no change over a long period, and the authors successfully performed TSS.^3^ If possible, preoperative testicular function should be evaluated to determine if TSS is truly necessary. Simultaneous testicular sperm extraction should be considered for patients who wish to have children and have azoospermia in the preoperative semen analysis or who are expected to have decreased testicular function.^4^

## Conflict of interest

The author declares no conflict of interest.
